# Fetal growth restriction, low birth weight, and preterm birth: Effects of active or passive smoking evaluated by maternal expired CO at delivery, impacts of cessation at different trimesters

**DOI:** 10.18332/tid/152111

**Published:** 2022-08-26

**Authors:** Conchita Delcroix-Gomez, Michel-Henri Delcroix, Amal Jamee, Tristan Gauthier, Pierre Marquet, Yves Aubard

**Affiliations:** 1 Service d'Obstétrique et de Gynécologie, Pôle Femme et Enfant, Centre Hospitalier d'Arras, Arras, France; 2 Etablissement Public de Santé Mentale des Flandres, Maternité Sans Tabac, Association Périnatalité Recherche Information, Bailleul, France; 3 University of Palestine, Gaza, Gaza Strip, Palestine; 4 Service d'Obstétrique et de Gynécologie, Hôpital de la Mère et de l’Enfant, Centre Hospitalier Universitaire de Limoges, Limoges, France; 5 Service de Pharmacologie, Toxicologie et Pharmacovigilance, Centre Hospitalier Universitaire de Limoges, Limoges, France

**Keywords:** smoking, pregnancy, expired carbon monoxide, fetal growth restriction, preterm birth

## Abstract

**INTRODUCTION:**

The objectives of this study were to evaluate the effect of cessation of active smoking during the 1st, 2nd, and 3rd trimesters of pregnancy on the risk of reduced birth weight and prematurity using an exhaled carbon monoxide biomarker with a cut-off value ≥3 ppm as well as the effects of passive smoking.

**METHODS:**

This was a multicenter prospective cohort study involving pregnant smokers and non-smokers. Pregnant smokers were identified at the first prenatal visit before 15 weeks of amenorrhea by the number of cigarettes smoked per day and by the carbon monoxide breath test. Women were classified into 6 groups: non-smokers, passive smokers, first trimester cessation, second trimester cessation, third trimester cessation, and smoking throughout pregnancy. Smoking cessation was defined if the pregnant woman reported quitting smoking and if she achieved an exhaled CO level of <3 ppm. The association between smoking cessation and fetal growth restriction or prematurity was assessed by multivariate logistic regression. Passive smoking was defined for non-smoking women on declarative smoking status and exhaled CO ≥3 ppm. The association between passive smoking and fetal growth restriction or prematurity was assessed by multivariate logistic regression.

**RESULTS:**

The number of patients included was 5244. The incidence of fetal growth restriction below the 10th percentile was 10.6%, 12.1%, 8.5%, 9.1%, 21.1%, and 22.9%, respectively, for the non-smoking, passive smoking, first, second, third trimester cessation, and full-pregnancy smoking, groups. The risk of FGR compared to non-smokers was OR=2.3 (95% CI: 1.18–4.30, p=0.014) for patients who quit smoking in the third trimester, OR=2.5 (95% CI: 2.03–3.12, p<0.001) for women who smoked throughout pregnancy. After logistic regression, FGR (AOR=1.9; 95% CI: 0.96–3.82) for women who quit smoking in the 3rd trimester (AOR=1.8; 95% CI: 1.38–2.31, p<0.001). The risk of FGR <5th percentile was AOR=1.96 (95% CI: 1.36–2.48, p<0.001).

**CONCLUSIONS:**

Active or passive smoking during pregnancy is associated with an increased risk of intrauterine growth restriction and low birth weight. Cessation in the 1st and 2nd trimester reduces the risk of intrauterine growth restriction or low birth weight. Passive smoking has a deleterious impact on fetal development, intermediate to that of active smoking.

## INTRODUCTION

In France, while cigarette consumption has decreased among men over the past 20 years, it has remained stable among young women. According to the ESCAPAD study (*Survey on Health and Consumption during the call to arms*), girls now smoke as much as boys^1^. Perinatal surveys from 2010 to 2016 show that maternal smoking is a public health problem as 30.6% of women smoke before pregnancy and 17.1% of these smokers continue to smoke at least one cigarette per day in the third trimester of pregnancy^2^. According to 2018 figures from Europeristat, France is the European country where women smoke the most before pregnancy, and ranks second for pregnant women who smoke in the third trimester of pregnancy^3^.

Pregnancy is the most favorable time to stop smoking, because of the expectation of the child and the organization of prenatal follow-up^4^. However, complete cessation may be difficult to achieve in women with a strong nicotine dependence. The last expert report of the CNGOF-SFT (*Collège National des Gynécologues ou Obstétriciens Français - Société Francophone de Tabacologie*) evaluated the association between active smoking and the occurrence of complications during pregnancy^5^.

Subsequent Surgeon General reports, US Department of Health and Human Services (USDHHS 2004, 2010, 2014, 2020)^6^ have identified causal links between active smoking and other adverse effects on women’s reproductive health, including decreased female fertility, pregnancy complications such as: ectopic pregnancy, spontaneous abortion, placental abruption, placenta previa, premature rupture of membranes (PROM), preterm delivery, birth defects, low birth weight (LBW), and fetal growth restriction (FGR)^7^.

According to the CNGOF, small-for-gestational-age (SGA) is defined by a weight (estimated fetal weight *in utero* or birth weight) below the 10th percentile. Small for gestational age is said to be severe if it is below the 3rd percentile. Fetal growth restriction (FGR) or intrauterine growth retardation (IUGR) generally corresponds to SGA associated with signs of abnormal growth (with or without abnormal uterine and/or umbilical Doppler): growth arrest or growth rate lag measured longitudinally (at least two measurements 3 weeks apart)^8^. The simplest measure to evaluate this is low birth weight in relation to gestational age; the threshold most often used is less than the 10th percentile of the AUDIPOG (*Association des Utilisateurs des Dossiers Informatisés en Pédiatrie, Obstétrique et Gynécologie*) neonatal curve, adjusted on four maternal parameters (age, weight, height, parity) and three neonatal parameters (gestational age, sex, birth rank). According to the 2010 perinatal survey in France, the proportion of FGR was 8.9% of all live births and the proportion of children weighing <2500 g was 6.3%^9,10^.

According to the latest report from the Surgeon General (US Department of Health and Human Services 2020)^6^, there is insufficient evidence to infer that the risk of low gestational age weight in women who quit smoking before or in early pregnancy does not differ from that of non-smokers.

Studies that have observed the impact of smoking during pregnancy and the influence of the date of cessation on the risk of FGR and low birth weight were not based on validation of cessation by a biomarker. Gaps in the literature concern how to assess smoking exposure, active or passive, and the impact of these on birth weight, with an easy-to-use biomarker such as exhaled carbon monoxide (CO) with mostly a validated cut-off value ≥3 ppm^11^.

Most studies in fact, assessed only active smoking and only from the woman’s self-report of her daily number of cigarettes smoked, most often categorized as either 1–10 cigs/day, ≥10 cigs/day, or 1–9 cigs/day, 10–19 cigs/day, and >20 cigs/day^12-14^.

The study of Secker-Walker et al.^15^ had shown the effect of CO from tobacco smoke on the reduction of birth weight and not specifically on fetal growth restriction. In a previous study, we showed that birth weight was dose dependent and significantly decreased when maternal exhaled CO increased with a reduction in mean birth weight of 755 g when maternal exhaled CO was >20 ppm compared to maternal exhaled CO <6 ppm^16^. Two previous studies have assessed the association between smoking or cessation of smoking and risk of GRF without biomarker validation and without consideration of passive smoking^17,18^.

The negative impact of passive smoking reported in several studies on low birth weight (<2500 g) has been mostly declarative without biomarker confirmation^19-21^.

The relevance of the exhaled CO biomarker to assess intrapartum smoking has been reported in the last two decades with cut-off values decreasing from 9 ppm^22^, to 5 ppm^16^, then 4 ppm^23,24^, and finally to 3 ppm^25^.

The objectives of this study were to evaluate the effect of cessation of active smoking during the 1st, 2nd, and 3rd trimesters of pregnancy on the risk of reduced birth weight and prematurity using an exhaled carbon monoxide biomarker with a cut-off value ≥3 ppm as well as the effects of passive smoking.

## METHODS

**Figure 1:**
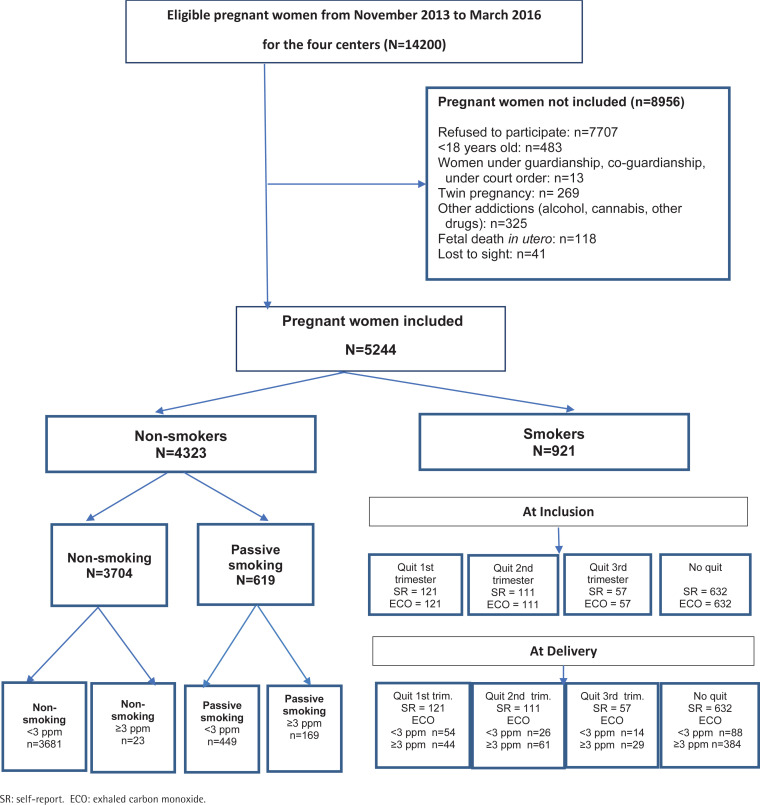
Flowchart of study in Limoges, Arras, Châteaudun, Neufchâteau, France, November 2013 to March 2016

This is a prospective, multicenter cohort study. Data were collected between November 2013 and January 2016 in four French maternity hospitals: Centre Hospitalier Universitaire (CHU) de Limoges (type III), Centre Hospitalier d'Arras (type III), Centre Hospitalier de Châteaudun (type I), and Centre Hospitalier de Neufchâteau (type I). This study was classified as low-risk and was approved by the ethics committee of the CHU of Limoges in March 2013. A flow chart of participant selection is given in Figure 1.

The inclusion criteria were: pregnant women, smokers or not, aged >18 years, single pregnancy, French-speaking, consulting at the latest at 15 weeks of amenorrhea (WA) for prenatal follow-up and giving birth in one of the study maternity units, benefiting from medical and social coverage, and having signed the consent to participate in the study.

The criteria for non-inclusion were: pregnant women refusing to participate in the study, women aged <18 years, women under guardianship, curatorship, or under court order, women with alcohol or cannabis dependence, women with other drug dependence, non-monofetal pregnancies, fetal deaths *in utero*, patients who were not followed throughout their pregnancy in one of the study maternity units, and pregnant women declining to participate in the study.

### Self-reported smoking status

Smoking exposure was assessed during the 7 mandatory prenatal visits. The self-reported smoking status of pregnant women was defined by the following questions: ‘Do you smoke?’, if yes: ‘did you smoke before your pregnancy?’ and ‘how many cigarettes did you smoke per day?’ If no, ‘Are you a non-smoker or did you quit smoking during your pregnancy?’, if yes: ‘at what point in your pregnancy did you quit smoking?’. Finally, for non-smokers, the question ‘Are you exposed to secondhand smoke from your spouse or relatives?’ was also filled in with the result of the exhaled CO measurement ≥3 ppm, which classified these women as passive smokers.

Smoking exposure was assessed at inclusion on the basis of self-reported number of cigarettes per day (1–10 cigs/day or >10 cigs/day) and exhaled CO biomarker, and then at each prenatal visit (2nd, 3rd, 4th, 5th, 6th, 7th, 8th, and 9th months of pregnancy) and at delivery. Information on self-reported smoking was collected at each prenatal visit.

Smoking cessation was confirmed when the woman attended one of these visits, declared that she had quit smoking, and presented an exhaled CO result <3 ppm: for women who responded that they had quit before 15 WA, cessation in the 1st trimester was validated; for women who responded that they had quit between 15 and 28 weeks of amenorrhea, cessation was validated in the 2nd trimester; for women who quit after 28 WA and before their delivery, cessation was validated in the 3rd trimester. Pregnant non-smoker status without exposure to passive smoking was validated if she reported at inclusion never having smoked and having an exhaled CO result <3 ppm. Pregnant non-smoker but exposed to passive smoking status was validated if she reported being exposed to her spouse’s passive smoking and having an exhaled CO result ≥3 ppm^25^. All information was recorded in the medical record at each prenatal visit.

### Threshold value for expired CO

The literature is divergent on the threshold value of exhaled CO (ppm) to distinguish pregnant smokers from non-smokers. The study of Bailey et al.^23^ had performed an ROC curve, showing the highest specificity and sensitivity for the cut-off point at >4 ppm^23^. Reynolds et al.^25^, after a sensitivity and specificity analysis, identified a cut-off value at 3 ppm to differentiate smokers from non-smokers, with a sensitivity 0.86. Based on the results of the latter study, we selected the threshold of ≥3 ppm to differentiate non-smoking pregnant women from smoking women or non-smokers exposed to passive smoking, and analyzed the association between active or passive smoking thus assessed and the risks of FGR, low birth weight, and prematurity.

### Exhaled CO measurement technique

The measurement of exhaled CO is a method for assessing the smoking status of pregnant women that has already been validated^25^. The technique for measuring CO in exhaled air is simple and consists in asking the pregnant woman, after a deep inspiration and an apnea of 10 seconds (in order to obtain an equilibrium between blood and alveolar CO levels), to exhale for as long as possible by clamping her lips on the mouthpiece of the CO analyzer. The chemical electrode in contact with the exhaled alveolar air measures CO. The CO analyzers used were of two brands: Eolys^®^, (Micro Medical Ltd. Rochester, Kent, UK) or F.I.M. Medical. They were regularly checked by the biomedical department of the hospitals. All health professionals in the prenatal sector were trained to measure exhaled CO in the context of prenatal care. This measurement was performed systematically for each patient participating in the study at inclusion for smokers and non-smokers, at each prenatal visit for smokers and in the delivery room for smokers and non-smokers. The result, immediate and expressed as the concentration of CO in exhaled alveolar air in parts per million (ppm), was recorded in the patient’s computerized or paper chart. A result ≥3 ppm verified exposure, in the 4 hours preceding the measurement, to tobacco smoke, through active or passive smoking.

### Identification of psychosocial vulnerabilities

Social and/or psychological vulnerabilities were identified during the early prenatal interview (EPP). This individual (and/or couple) interview is systematically proposed to each pregnant woman, during the 4th month of pregnancy, in order to prepare with her the best possible conditions for the birth of the child^26^. This face-to-face interview lasted 45 minutes. The indicators of vulnerability, collected using an assessment questionnaire or the EPP interview guide, were as follows: a minor (aged <18 years), poorly monitored pregnancy, history of secret birth or psychiatric pathology, intellectual disability, placement with the Social Assistance for Children (SAC), complex medico-psychosocial situation, domestic violence, lack of family and social support, lack of income or irregular income, lack of medical coverage or health insurance, consumption of toxic products (tobacco, cannabis, alcohol), drug addiction of the spouse, obstetrical history (denial of pregnancy, late diagnosis of pregnancy, fetal death *in utero*, medical termination of pregnancy, close pregnancy <6 months, parity >5, pregnancy continued after request for voluntary termination of pregnancy, and history of sudden infant death syndrome. The indicators of vulnerability were scored by the midwife or obstetrician-gynecologist from 0 (no significant vulnerability identified) to 4 (vulnerability clearly identified)^26^. The result was noted in each patient’s computerized or paper chart. For our study, we simplified the result of the variable ‘medico-psychosocial vulnerability’, with 0 and 1=no, and 2, 3 and 4=yes.

### The data collected

Data were collected by individual interview at the first consultation and at each monthly prenatal consultation until delivery and from the computerized or paper medical record of each patient: sociodemographic characteristics (age, parity, body mass index, education level, professional activity), existence of social or psychological vulnerability, self-reported smoking status (non-smoker, smoker at the beginning of pregnancy), number of cigarettes smoked per day, smoking behavior (whether or not stopped smoking during the 1st, 2nd, or 3rd trimester, or continued throughout the pregnancy), and for non-smokers whether or not they are exposed to passive smoking by their spouse or relatives. For each woman, the declarative smoking status data were combined with the measurement of exhaled CO. Gestational age, in weeks of amenorrhea (WA), was calculated from the date of the last menstrual period and a dating ultrasound.

The data collected in the medical record were also the mode of delivery, the occurrence or not of pathologies such as prematurity, comorbidities (pre-eclampsia, gestational diabetes, anemia), characteristics of the newborn (gestational age, birth weight in percentile according to the AUDIPOG curve). The following information was collected: birth weight, low birth weight (<2500 g) and very low birth weight (<1500 g), fetal growth restriction (FGR) <10th percentile and <5th percentile, sex, APGAR score < or >7 at 1 minute, and choice of breastfeeding type.

Based on the results of Reynolds et al.^25^ , we chose exhaled CO ≥3ppm as the cut-off value to differentiate exposure, from non-smoking. We classified our population of pregnant women, combining the exhaled CO biomarker result and self-reported status, into six groups: non-smokers (never smoked and <3ppm), non-smokers exposed to passive smoking (exposed to passive smoking and exhaled CO ≥3ppm), 1st, 2nd, and 3rd trimester smoking cessation (smoking cessation associated with exhaled CO <3 ppm), and smokers throughout pregnancy (1–10 cigs/day or >10 cigs/day with exhaled CO ≥3ppm).

### Covariates measured

Age was divided into 18–25 years, 26–35 years, and ≥36 years; education level categorized into middle school or less, high school, university, and postgraduate; occupation: employed, unemployed; smoking behavior into number of cigarettes per day: 1–10 and >10; exhaled CO <3 ppm and ≥3 ppm; parity in primiparous, second para, ≥ third para; body mass index (BMI, kg/m^2^) categorized as: underweight <18.5, normal 18.5–24.9, overweight 25–29.9, obese ≥30; for comorbidities: preeclampsia defined as hypertension after 20 WA (systolic blood pressure >140 mmHg and diastolic >90 mmHg), associated with proteinuria >0.3 g/24h; type 1 or type 2 or gestational diabetes (fasting blood glucose ≥0.92 g/L and/or after orally induced hyperglycemia: 75 g glucose, at 1 hour ≥1.80 g/L and/or at 2 hours ≥1.53 g/L); and anemia: hemoglobin <10.5 g/L. Prematurity was defined as delivery before 37 WA. Delivery modes were categorized into normal vaginal delivery, instrumental delivery (vacuum, forceps), and cesarean section. Mean birth weight was calculated in grams with standard deviation; low birth weight was categorized into low birth weight <2500 g and <1500 g. Regarding Apgar score at one minute of life: only scores <7 were counted. Regarding FGR: two categories were retained, FGR <10th percentile, and FGR <5th percentile. Breastfeeding modes were categorized as breastfeeding, artificial feeding, or mixed breastfeeding.

### Calculation of the FGR

The calculation of the FGR was performed automatically by hospital software: this modeling was used in this study to calculate the individual 10th and 5th percentile weight limit, below which a newborn was considered to have an FGR <10th and <5th percentile^10^. This software used the AUDIPOG (Association des Utilisateurs des Dossiers Informatisés en Pédiatrie, Obstétrique et Gynécologie) neonatal curve, adjusted for four maternal parameters (age, weight, height, parity) and three neonatal parameters (gestational age, sex, birth rank).

### Statistical analysis

All data were analyzed with SPSS version 26.0 statistical software (Chicago, Illinois, USA). Descriptive statistics were used to assess study characteristics and proportions of delivery and pregnancy outcome data in the different smoking groups. Categorical variables were expressed as frequency distributions and cross-tabulations and were compared using the chi-squared test or Fisher’s exact test. Continuous data were expressed as mean and standard deviation and compared using ANOVA. Binary logistic regression was used to examine the association between fetal growth <10th percentile or <5th percentile and smoking status. Covariates with p<0.005 were included in an adjusted regression model. The final model was obtained using a stepwise inverse procedure. Results are presented as rates with associated p values, or odds ratios with 95% confidence intervals. A significance level of p<0.05 was considered. Birth weight took into account gestational age, sex, birth rank of the child, and maternal body mass index.

## RESULTS

A total of 5244 pregnant women were included over the period from November 2013 to March 2016 at the four participating centers: Center Hospitalier Universitaire (CHU) de Limoges (type III), Centre Hospitalier d'Arras (type III), Centre Hospitalier de Châteaudun (type I), and Centre Hospitalier de Neufchâteau (type I).

Table 1 shows that women who quit smoking in the 1st and 2nd trimesters were more likely to have a higher level of education, employment, and a slightly lower level of vulnerability than those who continued to smoke beyond the second trimester. Women who quit smoking in the third trimester or who smoked throughout their pregnancy had lower levels of education than non-smokers. They had higher psychosocial vulnerability than non-smokers. Pregnant women who had smoked throughout their pregnancy were more often heavier smokers (>10 cig/day) and more often had a lower body mass index (BMI) (<18.5 kg/m^2^) than non-smokers (p=0.003). The mean maternal age of smokers was significantly lower than that of non-smokers: 29.7 ±5.52 versus 30.2 ± 5.5 with p=0.005.

**Table 1:** Sociodemographic maternal characteristics in Limoges, Arras, Châteaudun, Neufchâteau, France, November 2013 to March 2016 (N=5244)

*Characteristics*	*Non-smoking (n=3704; 70.6%) n (%)*	*Passive smoking (n=619; 11.8%) n (%)*	*Quit first trimester (n=121; 2.3%) n (%)*	*Quit second trimester (n=111; 2.1%) n (%)*	*Quit third trimester (n=57; 1.1%) n (%)*	*Smoking through pregnancy (n=632; 12.1) n (%)*
**Mean maternal age**^†^, mean ± SD	30.2 ± 5.49	30.1 ± 5.73	30.8 ± 5.32	29.5 ± 4.44	27.9 ± 4.51	29.7 ± 5.52
**Age** (years)						
18–25	739 (20.2)	133 (21.5)	18 (14.9)	18 (16.2)	19 (33.3)	135 (21.4)
26–35	2342 (63.2)	382 (61.7)	80 (66.1)	85 (76.6)	36 (63.2)	399 (63.1)
≥36	623 (16.8)	104 (16.8)	23 (19.0)	8 (7.2)	2 (3.5)	98 (15.5)
**Education level**						
Middle school or less	647 (17.5)	163 (26.3)	11 (9.1)	3 (2.7)	23 (40.4)	231 (36.6)
High school	1541 (41.6)	139 (22.5)	43 (35.5)	82 (73.9)	25 (43.9)	335 (53.0)
University and postgraduate	1516 (40.9)	317 (51.2)	67 (55.4)	26 (23.4)	9 (15.8)	66 (10.4)
**Occupation**						
Employed	2684 (72.5)	385 (62.2)	115 (95.0)	107 (96.4)	10 (17.5)	450 (71.0)
Unemployed	1020 (27.5)	234 (37.8)	6 (5.0)	4 (3.6)	47 (82.5)	182 (28.8)
**Vulnerability psychosocial**						
Yes	1447 (39.1)	198 (32.0)	93 (76.9)	98 (88.3)	52 (91.2)	589 (93.2)
No	2257 (60.9)	421 (68.0)	28 (23.1)	13 (11.7)	5 (8.8)	43 (6.8)
**Number of cigarettes/day**						
1–10	-	-	116 (95.9)	106 (95.5)	36 (63.2)	465 (73.6)
>10	-	-	5 (4.1)	5 (4.5)	21 (36.8)	167 (26.4)
**Parity**						
1	1569 (42.9)	286 (46.2)	78 (64.5)	55 (49.5)	24 (42.1)	345 (54.6)
2	1330 (35.9)	192 (31.0)	39 (32.2)	29 (26.1)	11 (19.3)	128 (20.3)
≥3	805 (21.7)	142 (22.8)	4 (3.3)	27 (24.3)	22 (38.6)	159 (25.2)
**Maternal comorbidity**						
BMI^††^, mean ± SD*	24.40 ± 4.94	23.65 ± 4.82	23.88 ± 5.08	23.65 ± 5.40	24.26 ± 5.07	24.06 ± 5.06
Underweight	240 (6.5)	67 (10.8)	14 (11.6)	11 (9.9)	5 (8.8)	74 (11.7)
Normal	2083 (56.2)	358 (57.8)	71 (58.7)	57 (51.4)	29 (50.9)	337 (53.3)
Overweight	852 (23.0)	124 (20.0)	15 (12.4)	24.9 (21.6)	13 (22.8)	144 (22.8)
Obesity	529 (14.3)	70 (11.3)	21 (17.4)	19 (17.1)	10 (17.5)	77 (12.2)
Diabetes	425 (11.5)	49 (7.9)	12 (9.9)	10 (9.0)	4 (7.0)	40 (6.3)
Pre-eclampsia	82 (2.2)	70 (11.3)	6 (5.0)	5 (4.5)	0 (0.)	21 (3.3)
Anemia <10.5 g/dL	15 (0.40)	4 (0.6)	6 (5.0)	0 (0.0)	6 (10.5)	12 (1.9)
No disease	2462 (66.5)	225 (36.3)	53 (43.8)	61 (55.0)	16 (28.1)	245 (38.8)

* Body mass index (BMI, kg/m^2^): underweight <18.5, normal 18.5–24.9, overweight 25.0–29.9, obese ≥30.

† Mean maternal age: difference between group was statistically significant p=0.005.

†† Body mass index mean: difference between group was statistically significant p=0.003.

Table 2 shows that the mean birth weight associated with smoking in each group was significantly lower than in the non-smoking group. Compared with non-smokers, women who quit smoking in the third trimester or who smoked throughout pregnancy had a higher incidence of low birth weight (<2500 g). Among women who quit smoking in the first and second trimesters, mean birth weight was significantly higher than among women who quit smoking in the third trimester or smoked throughout pregnancy, and the risk of FGR was close to that of non-smokers. Conversely, women who quit smoking in the third trimester or smoked throughout pregnancy had a significantly higher risk of FGR <10th percentile or <5th percentile, approximately twice that of non-smokers. Similarly, they had a rate of preterm delivery that was also twice as high as non-smokers. The mean birth weight of passive smokers was significantly lower (2849 ± 677 g) than that of non-smokers (3331 ± 418 g). The mean birth weight of passive smokers was slightly higher than that of women who quit in the third trimester or smoked throughout the pregnancy (2805 g or 2815 g, respectively). The passive smoker group had a proportion of 21.5% of newborns with a weight <2500 g, which was three times higher than that of non-smokers and slightly lower than that of women who had stopped smoking in the third trimester or who had smoked throughout the pregnancy (22.8%). The passive smoker group had 16.3% of preterm infants, twice the proportion of non-smokers and slightly lower than women who quit smoking in the third trimester or smoked throughout pregnancy (17.5% and 17.2%, respectively).

**Table 2:** Maternal and birth outcomes by smoking status in Limoges, Arras, Châteaudun, Neufchâteau, France, November 2013 to March 2016 (N=5244)

	*Non-smoking (n=3704; 70.6%) n (%)*	*Passive smoking (n=619; 11.8%) n (%)*	*Quit first trimester (n=121; 2.3%) n (%)*	*Quit second trimester (n=111; 2.1%) n (%)*	*Quit third trimester (n=57; 1.1%) n (%)*	*Smoking through pregnancy (n=632; 12.1) n (%)*
**Maternal**						
Gestational age (WA), mean ± SD	39.7 ± 1.3	36.3 ± 4.3	38.3 ± 4.1	38.2 ± 2.5	37.8 ± 1.7	37.9 ± 4.3
**Type of delivery**						
Vaginal	2516 (67.9)	396 (64)	83 (68.6)	84 (75.7)	43 (75.4)	403 (63.8)
Instrumental	730 (19.7)	127 (20.5)	27 (22.3)	13 (11.7)	10 (17.5)	156 (24.7)
Cesarean	458 (12.4)	96 (15.5)	11 (9.1)	14 (12.6)	4 (7.0)	73 (11.6)
**Newborn**						
BW (g), mean ± SD*	3331 ± 418	2849 ± 677	3062 ± 735	2950 ± 513	2805 ± 429	2815 ± 735
LBW (<2500 g)	251 (6.8)	133 (21.5)	17 (14.0)	17 (12.6)	13 (22.8)	144 (22.8)
ELBW (<1500 g)	46 (1.2)	39 (6.3)	6 (5.0)	1 (0.9)	1 (1.8)	43 (6.8)
Apgar score <7 to 1 min	273 (7.4)	100 (16.2)	6 (5.0)	1 (0.9)	0 (0.0)	26 (4.1)
Preterm birth <37 WA	269 (7.3)	101 (16.3)	15 (12.7)	13 (11.8)	10 (17.5)	107 (17.2)
FGR^†^ (<10th percentile)	392 (10.6)	75 (12.1)	10 (8.5)	10 (9.1)	12 (21.1)	141 (22.9)
FGR^††^ (<5th percentile)	196 (5.3)	36 (5.8)	4 (3.4)	2 (1.8)	5 (8.8)	63 (10.2)
**Gender**						
Male	1933 (52.2)	320 (51.7)	65 (53.7)	52 (46.8)	30 (52.6)	317 (50.2)
Female	1771 (47.8)	299 (48.3)	56 (46.3)	59 (53.2)	27 (47.4)	315 (49.8)
**Chosen feeding**						
Breastfeeding	2165 (58.5)	344 (55.6)	32 (26.4)	33 (29.7)	19 (33.3)	213 (33.7)
Artificial feeding	1303 (35.2)	234 (37.8)	84 (69.4)	76 (68.5)	38 (66.7)	380 (60.1)
Mixed breastfeeding	236 (6.4)	40 (6.5)	2 (1.7)	1 (0.9)	0 (0.0)	22 (3.5)

WA: weeks of amenorrhea.

* The average birth weight took into account gestational age, sex, birth rank of the child, and maternal body mass index. BW: birth weight; LBW: low birth weight; ELBW: extreme low birth weight.

† FGR: fetal growth restriction <10 percentile;

†† FGR: fetal growth restriction <5 percentile. The difference between groups was statistically significant p<0.001.

Our results also showed that the average length of pregnancy was impacted by smoking: women who were passive smokers had a shorter average length of pregnancy compared to non-smokers or compared to women who stopped smoking in the 1st and 2nd trimesters of pregnancy or compared to women who stopped smoking in the 3rd trimester or who continued to smoke throughout their pregnancy. The difference between groups was statistically significant p<0.001.

Table 3 shows that apart from smoking, low education significantly increased the risk of having a child with FGR <10th percentile and <5th percentile. Lack of employment and psychosocial vulnerability also increased the risk of having a child with FGR <10th percentile, but not at the 5th percentile.

**Table 3:** Maternal characteristics by fetal growth restriction status in Limoges, Arras, Châteaudun, Neufchâteau, France, November 2013 to March 2016 (N=5244)

*Characteristics*	*Fetal growth restriction < 10th percentile*			*Fetal growth restriction < 5th percentile*		
	*n (%)*	*OR (95% CI)*	*p*	*n (%)*	*OR (95% CI)*	*p*
**Age** (years)						
18–25	135 (21.1)	1.1 (0.87–1.30)	0.567	61 (19.9)	0.90 (0.73–1.31)	0.891
26–35	405 (63.3)	1.1 (0.84–1.19)	0.962	201 (65.7)	1.1 (0.87–1.42)	0.385
≥36	100 (15.6)	0.9 (0.74–1.18)	0.576	44 (14.4)	0.85 (0.61–1.18)	0.327
**Education level**						
Middle school or less	285 (44.5)	3.8 (3.25–4.60)	<0.001	213 (69.6)	10.8 (8.37–13.9)	<0.001
High school	218 (34.1	0.7 (0.59–0.84)	<0.001	72 (23.5)	0.4 (0.32–0.55)	<0.001
University and postgraduate	137 (21.4)	0.4 (0.32–0.48)	<0.001	21 (6.9)	0.1 (0.07–0.17)	<0.001
**Occupation**						
Unemployed	206 (32.2)	1.2 (1.02–1.46)	0.028	100 (32.7)	1.2 (0.96–1.56)	0.096
Employed	434 (67.8)	0.8 (0.69–0.98)		206 (67.3)	0.8 (0.63–1.04)	0.096
**Psychosocial vulnerabilities**						
Yes	330 (51.6)	1.2 (1.04–1.45)	0.014	152 (49.7)	1.1 (0.89–1.41)	0.340
No	310 (48.4)	0.8 (0.70–0.96)		154 (50.3)	0.9 (0.71–1.13)	0.340
**Parity**						
1 parity	298 (46.6)	1.1 (0.92–1.28)	0.362	137 (44.8)	1.0 (0.79–1.26)	0.967
2 parity	185 (28.9)	0.8 (0.68–1.00)	0.019	87 (28.4)	0.8 (0.62–1.03)	0.080
≥3 parity	157 (24.5)	1.2 (0.96–1.42)	0.116	82 (26.8)	1.3 (1.01–1.70)	0.042
Maternal BMI*						
Underweight	50 (7.8)	1.0 (0.73–1.36)	0.984	32 (10.5)	1.5 (0.96–2.06)	0.078
Normal	353 (55.2)	1.0 (0.81–1.14)	0.641	168 (54.9)	1.0 (0.76–1.20)	0.687
Overweight	145 (22.7)	1.0 (0.84–1.24)	0.841	66 (21.6)	1.0 (0.72–1.26)	0.736
Obesity	92 (14.4)	1.0 (0.83–1.34)	0.657	40 (13.1)	0.9 (0.66–1.32)	0.701
**Maternal comorbidity**						
No disease	321 (50.2)	0.7 (0.57–0.79)	<0.001	159 (52.0)	0.8 (0.61–0.95)	0.015
Diabetes	45 (7.0)	0.6 (0.46–0.86)	0.004	17 (5.6)	0.5 (0.30–0.81)	0.005
Pre-eclampsia	33 (5.2)	1.6 (1.08–2.35)	0.017	14 (4.6)	1.3 (0.77–2.34)	0.304
Anemia <10.5 g/dL	5 (0.8)	1.1 (0.42–2.70)	0.900	3 (1.0)	1.2 (0.37–3.92)	0.754

* Body mass index (BMI, kg/m^2^): underweight: <18.5, normal 18.5–24.9, overweight: 25.0–29.9, obese: ≥30.

Logistic regression was used to estimate the adjusted odds ratio of an FGR <10th and <5th percentile, associated with smoking cessation or smoking throughout pregnancy, adjusting for maternal age, parity, education level, occupational status at the start of pregnancy, and maternal comorbidities (underweight, overweight, obesity, diabetes, pre-eclampsia, anemia) (Table 4). The results in Table 4 show a significant increase in the risk of FGR <10th percentile for women who quit smoking only in the third trimester or who smoked throughout pregnancy (OR=2.3; 95% CI: 1.18–4.30, p<0.014, and OR=2.5; 95% CI: 2.03–3.12, p<0.001, respectively). After adjustment for maternal characteristics, this risk was significant only for women who smoked throughout pregnancy (AOR=1.8; 95% CI: 1.38–2.31, p<0.001). The crude risk of having an FGR below the 5th percentile was significantly increased for women who smoked throughout pregnancy. After adjustment for maternal comorbidities, this risk was significant (AOR=1.96; 95% CI: 1.36–2.48, p<0.001).

**Table 4:** Logistic regression for fetal growth restriction by smoking status in Limoges, Arras, Châteaudun, Neufchâteau, France, November 2013 to March 2016 (N=5244)

	*Fetal growth restriction < 10th percentile*				*Fetal growth restriction < 5th percentile*			
	*OR (95% CI)*	*p*	*AOR (95% CI)*	*p*	*OR (95% CI)*	*p*	*AOR (95% CI)*	*p*
**Smoking status**								
Non-smoking (Ref.)	1	-	1	-	1	-	1	-
Passive smoking	1.2 (0.90–1.52)	0.250	1.0 (0.72–1.28)	0.774	1.1 (0.77–1.60)	0.586	0.8 (0.54–1.24)	0.336
Quit, 1st trimester	0.8 (0.41–1.51)	0.463	0.9 (0.45–1.77)	0.751	0.6 (0.23–1.72)	0.365	0.9 (0.31–2.60)	0.849
Quit, 2nd trimester	0.8 (0.44–1.63)	0.616	1.0 (0.51–1.99)	0.975	0.3 (0.08–1.35)	0.124	0.5 (0.12–2.16)	0.367
Quit, 3rd trimester	2.3 (1.18–4.30)	0.014	1.9 (0.96–3.82)	0.064	1.7 (0.68–4.36)	0.250	0.9 (0.32–2.27)	0.751
Smoked through pregnancy	2.5 (2.03–3.12)	<0.001	1.8 (1.38–2.31)	<0.001	2.0 (1.52–2.75)	<0.001	1.96 (1.36–2.48)	<0.001

Smoking status in category of fetal growth restriction <10th percentile was adjusted for education level, occupation, vulnerability psychosocial and maternal comorbidities. Smoking status in category of fetal growth restriction <5th percentile was adjusted for maternal comorbidities.

In contrast, the results confirmed that smoking cessation before the beginning of the third trimester eliminated the excess risk of FGR. Table 5 shows the relationship between maternal exhaled CO levels (<3 ppm or ≥3 ppm) and smoking status, Apgar score <7 at 1 minute, prematurity, and FGR <10th and <5th percentiles. Women who smoked were more likely to have an exhaled CO level ≥3 ppm (OR=52.2; 95% CI: 41.35–65.99, p<0.001 for <10 cigs/day) and the risk doubled if the woman smoked ≥10 cigs/day (OR=130.0; 95% CI: 80.19–211.04, p<0.001).

**Table 5:** Smoking status and neonatal outcomes analyzed by maternal expired CO levels <3 ppm and ≥3 ppm at delivery in Limoges, Arras, Châteaudun, Neufchâteau, France, November 2013 to March 2016

	*<3 ppm n (%)*	*≥3 ppm n (%)*	*OR (95% CI)*	*p*
**Smoking status**				
Non-smoking	3681 (85.4)	23 (3.2)	0.006 (0.004–0.009)	<0.001
Passive smoking	449 (10.4)	169 (23.8)	2.7 (2.2–3.28)	<0.001
Quit, 1st trimester	54 (1.3)	44 (6.2)	5.2 (3.47–7.82)	<0.001
Quit, 2nd trimester	26 (0.6)	61 (8.6)	15.5 (9.72–24.70)	<0.001
Quit, 3rd trimester	14 (0.3)	29 (4.1)	13.1 (6.87–24.87)	<0.001
Smoking through pregnancy	88 (2.0)	384 (54.1)	56.5 (43.70–73.15)	<0.001
**Number of cigarettes/day**				
1–10 cigarettes/day	161 (3.7)	391 (55.1)	52.2 (41.35–65.99)	<0.001
>10 cigarettes/day	21 (0.5)	127 (17.9)	130.0 (80.19–211.04)	<0.001
**Apgar score**				
<7 to 1 min	330 (7.7)	76 (10.7)	1.4 (1.11–1.88)	0.006
**Prematurity**				
Preterm birth <37 WA	254 (5.9)	246 (35.3)	8.7 (7.14–10.67)	<0.001
**Fetal growth restriction**				
FGR (<10th percentile)	448 (10.4)	162 (23.4)	2.6 (2.15–3.22)	<0.001
FGR (<5th percentile)	223 (5.2)	67 (9.6)	2.0 (1.48–2.62)	<0.001

WA: weeks of amenorrhea. ECO: exhaled carbon monoxide. The p-value compares women with ECO levels <3ppm to women with ECO levels ≥3 ppm.

The incidence of LBW <10th percentile was greater in women with exhaled CO ≥3 ppm, (OR=2.6; 95% CI: 2.15-3.22, p<0.001). The incidence of preterm births was also higher in women with exhaled CO ≥3 ppm (OR=8.7; 95% CI: 7.14–10.67, p<0.001). In our study, 23.8% of women who reported being non-smokers were exposed to passive smoking, had an exposure measurement ≥3 ppm.

In addition, our results also show that pregnant smokers who quit smoking in the 1st, 2nd, or 3rd trimester could also be exposed to passive smoking:

1. Smoking cessation 1st trimester: n=44 (6.2%) had exhaled CO ≥3ppm at delivery despite cessation;
2. Smoking cessation 2nd trimester: n= 61 (8.6%) had exhaled CO ≥3ppm at delivery despite cessation; and
3. Smoking cessation 3rd trimester: n= 29 (4.1%) had exhaled CO ≥3ppm at delivery despite cessation.

Our results show high ORs for prematurity, FGR <10th percentile or <5th percentile, when maternal CO exhaled at delivery was ≥3 ppm. Finally, the incidence of an APGAR score <7 at 1 minute was moderately but significantly increased in neonates of mothers with expired CO ≥3 ppm.

## DISCUSSION

### Effects of smoking cessation

Our results show the beneficial impact of stopping active smoking in the first and second trimester on mean weight and FGR risk compared with women who smoked throughout the pregnancy. Indeed, the incidence of FGR <10th percentile was 8.5% for women who smoked and stopped in the first trimester and 9.1% for women who stopped in the second trimester, whereas it was 22.9 % for women who smoked throughout their pregnancy. We also showed that compared to non-smoking pregnant women, women who quit smoking before 3rd trimester did not significantly increase their risk of FGR <10th percentile or <5th percentile. In our study, we found that infants of mothers who quit smoking in the 1st and 2nd trimesters were less likely to have LBW <2500 g, extreme LBW <1500 g, preterm birth, FGR <10th or <5th percentile, than infants of mothers who quit smoking in the 3rd trimester or who continued to smoke throughout pregnancy. These results are comparable to those of other studies.

In women who quit smoking in the 1st and 2nd trimesters, the proportions of low birth weight <2500 g were significantly lower than in women who had quit in the 3rd trimester or smoked throughout the pregnancy. Similarly, quitting in the 1st and 2nd trimester of pregnancy decreased the risk of preterm delivery compared to those who quit in the 3rd trimester or smoked throughout the pregnancy.

Our study differs from other studies in the assessment of smoking exposure, active or passive, by using a biomarker, exhaled CO, based on a threshold value of smoking exposure ≥3 ppm (sensitivity of 0.89)^25^ associated with the reported number of cigarettes smoked per day. It is from this improved assessment of smoking exposure, active or passive, that the association between it and the occurrence of FGR, low birth weight, and prematurity was measured. Our results clearly show that self-reported smoking status during pregnancy alone was not sufficient to define the risk of exposure to active or passive smoking: the measurement of exhaled CO allowed better detection and verification of passive smoking in non-smokers or in women who reported to have stopped smoking in the 1st, 2nd, and 3rd trimester of pregnancy.

Our results are consistent with those of previous studies that have examined the risk of low birth weight and preterm delivery according to the trimester of cessation^11,17,27^. The study by Baba et al.^18^ showed that active maternal smoking increased the risk of low birth weight <2500 g by 26% if women smoked at the beginning of pregnancy (AOR=1.26; 95% CI: 1.09–1.46) and by a factor of 2.5 if they smoked throughout pregnancy (AOR=2.55; 95% CI: 2.43–2.67). According to the meta-analysis by Quelhas et al.13, active maternal smoking increased the risk of low birth weight by 69% (AOR=1.69; 95% CI: 1.59–1.79) for 1 to 10 cigarettes per day and increased this risk by 2.5-fold for more than 10 cigarettes per day (AOR=2.53; 95% CI: 2.31–2.78).

### Effects of passive smoking

Our study shows a very interesting result concerning passive smoking. A large proportion of non-smoking pregnant women were exposed to passive smoking (exhaled CO ≥3 ppm). Among their newborns, 21.5% had LBW, 6.3% extreme LBW, 16.3% of prematurity and 12.1% FGR.

The contribution of the present study was to systematically search, with the measurement of exhaled CO (≥3 ppm) for passive smoking, which is usually not searched for or poorly evaluated. Our study provides new information showing the adverse effects of passive smoking on preterm birth, LBW <2500 g, extreme LBW <1500 g or FGR, and the usefulness of systematic screening during pregnancy with exhaled CO (≥3ppm).

Our results also show the impact of active and passive smoking on preterm birth. Indeed, we found increasing proportions of preterm births from the passive smoking group to the active smoking group throughout the pregnancy had an eightfold higher incidence of preterm birth than non-smoking women. Our results evaluating the impact of passive smoking verified with an ECO ≥3 ppm are similar to those of Jaakkola et al.^28^, who found a 6-fold increase in risk (AOR=6.12; 95% CI: 1.31–28.70), by verifying the level of smoking with nicotine concentration in the hair (≥4 μg/g) as biomarker. They are also close to those of Raja et al.^29^, who showed a 4-fold increase in risk in case of heavy passive smoking (spouse smoked >20 cigs/day).

Studies evaluating tobacco exposure based on the reported number of cigarettes smoked per day had shown an association between the time of smoking cessation during pregnancy and the risk of low birth weight or preterm delivery^30-33^. Compared to these publications, the contribution of our study is to have evaluated the exposure to smoking, active or passive, by the use of a biomarker the exhaled CO with a threshold value of exposure >3 ppm (sensitivity of 0.89)^25^ associated with the declared number of cigarettes smoked per day. It is thus from this objective evaluation of the exposure to smoking, active or passive, that the link between it and the occurrence of FGR, low birth weight and prematurity was measured.

Indeed, an innovative aspect of our study is the use of the exhaled CO biomarker to assess the association between active maternal smoking, its cessation time in the 1st, 2nd and 3rd trimester of pregnancy and the risk of FGR, low birth weight, and prematurity. This biomarker is certainly used at the time of delivery but not only, since it was used at the time of inclusion in our prospective study. Of course, exhaled CO has limitations that are reduced if its measurement is repeated during pregnancy follow-up and combined with the declarative smoking status which is an indication of smoking exposure (amount, frequency, duration, time of pregnancy).

The validity of the measurement at the time of delivery is linked to the respect of good practices concerning exhaled CO which must be measured immediately upon admission on the day of delivery. More broadly, the diagnosis of exposure to active or passive smoking at the end of pregnancy with the measurement of exhaled CO (threshold value ≥3 ppm) makes it possible to propose an assessment, a management more adapted to the situation of each pregnant woman.

Our results thus clearly show that self-reported smoking status during pregnancy was not sufficient on its own to define the risk of exposure to active or passive smoking: the measurement of exhaled CO made it possible, in particular, to better detect and verify passive smoking in non-smokers or in women who had declared that they had stopped smoking during the 1st, 2nd and 3rd trimesters of pregnancy.

When the measurement of exhaled CO was systematically used, it made it possible to identify, within the limit of its half-life of 4 hours, the number of women really exposed to passive smoking (rate ≥3 ppm at delivery) in each of the analyzed groups. More than half of the pregnant women tested and smokers throughout their pregnancy had a level ≥3 ppm. A small number of pregnant women classified as non-smokers and not having declared passive smoking, had a level ≥3 ppm and were therefore subject to passive smoking not recorded by their declarative smoking status alone.

Thus, the measurement of exhaled CO allowed the detection of either passive smoking or continued active smoking in the 1st, 2nd, and 3rd trimester cessation groups for 134 women at delivery. This additional medical data may explain the significantly higher results of low birth weight observed in the ‘1st and 2nd trimester cessation groups’ compared to non-smokers.

Other studies on passive smoking, including a meta-analysis, have shown that there is a link between exposure to passive smoking, low birth weight, and FGR^34-36^.

### Strengths and limitations

Our study has several strengths, including a high number and percentage of pregnant smokers assessed with the exhaled CO biomarker ≥3 ppm. This is a prospective, multicenter, study on 4 sites of 5244 pregnant non-smokers and smokers that evaluated the effect of smoking cessation by trimester on the incidence of FGR and birth weight.

The contribution of the present study was to systematically search with exhaled CO measurement for passive smoking, which is usually not searched for or poorly assessed. Our study provides new information showing the adverse effects of passive smoking on the risk of low birth weight <2500 g, extreme LBW <1500 g, FGR, and the usefulness of systematically screening for the level of smoking exposure by measuring exhaled CO with a cut-off value ≥3 ppm. This study provides more comprehensive data, on the effect of the date of cessation (1st, 2nd, 3rd trimester of pregnancy) of active smoking, passive smoking, on the risks of FGR, low birth weight, and prematurity, assessed by the biomarker exhaled CO with a cut-off value ≥3 ppm.

Limitations of the study are the lack of information regarding history of FGR or prematurity and the lack of completeness of maternal exhaled CO measurement or the lack of paternal exhaled CO measurement. Finally, the relatively short half-life (4 hours) of the exhaled CO biomarker and the lack of use of another biomarker such as urinary cotinine to consolidate the patients smoking status or the use of anabasin in case of smoking resumption.

## CONCLUSIONS

Smoking cessation should occur as early as possible, ideally before 15 weeks of gestation, to bring the risk of FGR closer to that observed in non-smokers.

Our study shows the interest of measuring exhaled CO to detect and quantify the exposure of the fetus to active or passive smoking and to objectively evaluate the increased risk of FGR, low birth weight and prematurity according to this exposure. Our results confirm the interest of managing and evaluating smoking cessation throughout pregnancy so that each pregnant woman concerned has time to introduce a change in behavior beneficial to herself and her unborn child.

## Data Availability

The data supporting this research cannot be made available for privacy or other reasons. Each hospital remains the sole owner of its data.
